# Metabolic rate and hypoxia tolerance are affected by group interactions and sex in the fruit fly (*Drosophila melanogaster*): new data and a literature survey

**DOI:** 10.1242/bio.023994

**Published:** 2017-02-15

**Authors:** Warren Burggren, BriAnna M. Souder, Dao H. Ho

**Affiliations:** 1 DevelopmentalIntegrative Biology Group, Department of Biological Sciences, University of North Texas, Denton, TX 76203, USA; 2 Department of Clinical Investigation, Tripler Army Medical Center, Honolulu, HI 96859, USA

**Keywords:** *Drosophila*, Oxygen consumption, Sex, Social interaction, Respirometry techniques

## Abstract

Population density and associated behavioral adjustments are potentially important in regulating physiological performance in many animals. In *r*-selected species like the fruit fly (*Drosophila*), where population density rapidly shifts in unpredictable and unstable environments, density-dependent physiological adjustments may aid survival of individuals living in a social environment. Yet, how population density (and associated social behaviors) affects physiological functions like metabolism is poorly understood in insects. Additionally, insects often show marked sexual dimorphism (larger females). Thus, in this study on *D. melanogaster*, we characterized the effects of fly density and sex on both mass-specific routine oxygen consumption (*V̇O*_2_) and hypoxia tolerance (P_Crit_). Females had significantly lower routine *V̇O*_2_ (∼4 µl O_2_ mg^−1^ h^−1^) than males (∼6 µl O_2_ mg^−1^ h^−1^) at an average fly density of 28 flies·respirometer chamber^−1^. However, *V̇O*_2_ was inversely related to fly density in males, with *V̇O*_2_ ranging from 4 to 11 µl O_2_ mg^−1^ h^−1^ at a density of 10 and 40 flies·chamber^−1^, respectively (r^2^=0.58, *P*<0.001). Female flies showed a similar but less pronounced effect, with a *V̇O*_2_ of 4 and 7 µl O_2_ mg^−1^ h^−1^ at a density of 10 and 40 flies·chamber^−1^, respectively (r^2^=0.43, *P*<0.001). P_Crit_ (∼5.5 to 7.5 kPa) varied significantly with density in male (r^2^=0.50, *P*<0.01) but not female (r^2^=0.02, *P*>0.5) flies, with higher fly densities having a lower P_Crit_. An extensive survey of the literature on metabolism in fruit flies indicates that not all studies control for, or even report on, fly density and gender, both of which may affect metabolic measurements.

## Abstract (precis)

**Summary:** Technical advances allowing oxygen consumption measurement in individual fruit flies actually take them out of their normal highly social context, resulting in higher oxygen consumption rates than in natural groups.

## INTRODUCTION

The metabolic rate of the fruit fly *Drosophila* has been measured in numerous studies, with numerous intentions. Investigators have long used the fruit fly as a model for investigations of life span, and accordingly have measured metabolic rate in an often unsuccessful attempt to correlate metabolism to longevity (Arking et al., 1988; Baldal et al., 2006; Hulbert et al., 2004; Khazaeli et al., 2005; Melvin et al., 2007; Miquel et al., 1982; Partridge et al., 2005; Promislow and Haselkorn, 2002; Van Voorhies et al., 2003, 2004b). Metabolic rate in *Drosophila* has also been investigated in the context of specific genotypes (Hoekstra and Montooth, 2013; Jensen et al., 2014; Jumbo-Lucioni et al., 2010; Montooth et al., 2003; Stone et al., 2013), to reveal how genotype underpins specific metabolic phenotypes. Metabolic rate has also been measured for insight into how specific metabolic pathways affect energy metabolism (Barros et al., 1991; Isobe et al., 2013). The study of function and limitations of gas exchange by a tracheal system have also involved metabolic rate measurements (Klok et al., 2010; Merkey et al., 2011; Mölich et al., 2012). Finally, but not exhausting the list of reasons for measuring metabolic rate in *Drosophila*, a driver for such studies has been comparative physiological aspects including the effects of temperature or oxygen as stressors (Berrigan and Partridge, 1997; Folguera et al., 2010; Isobe et al., 2013; Lighton, 2007; Orr, 1925; Skandalis et al., 2011; Van Voorhies, 2009; Williams et al., 2004).

As varied as the rationale for metabolic rate measurements in *Drosophila* are the methodologies that have been employed. Most common has been the measurement of carbon dioxide emission or the consumption of oxygen, detected by respiratory gas sensors using open respirometry, closed respirometry and intermittent respirometry (for review see Van Voorhies et al., 2008). However, heat production/microcalorimetry (Hulbert et al., 2004; Piper et al., 2014; Van Voorhies et al., 2008) and even doubly labeled water techniques (Piper et al., 2014) have been employed. Until the last decade or so, most measurements were on small groups of flies, necessary to assemble sufficient biomass for accurate measurement of metabolic rate. In recent years, however, sensitivity of instrumentation has grown to the point that metabolic rate measurement on single flies is routine.

Not surprisingly, given the variety of approaches to measuring metabolic rate and the various protocols used, a nearly 200-fold variation exists in estimates of routine metabolic rate in *Drosophila.* The reasons for this variation have typically been attributed to differences in techniques (Van Voorhies et al., 2008). Yet, a myriad of biotic reasons can account for variability in comparative physiological studies, including sex, prandial state, time of day, and history including epigenetic influences (Burggren, 2014).

One factor of potentially great importance in metabolic rate in *Drosophila* is behavior, and especially social interaction between individuals. *Drosophila* is an insect with fairly stereotypic and well-studied social interactions, including numerous sex-specific behaviors (Mowrey and Portman, 2012; Portman, 2007; Schneider et al., 2016; Villella and Hall, 2008; Yamamoto et al., 2014). Given the nature of the behavioral interactions between individuals, one might anticipate that social interactions and the associated stereotypic behaviors in *Drosophila* might also directly or indirectly influence their metabolic rate. However, the technology-enabled trend to single fly analyses has, for better or worse, eliminated social interaction as a variable. Indeed, as Piper et al. (2014) so aptly commented, “(metabolic chamber) measurements require separating individuals from any social context, and may only poorly reflect the environment in which the animals normally live”. Yet, few previous studies have addressed the potentially complex relationships between social interaction and metabolic rate specifically in *Drosophila*.

Another poorly controlled variable in the measurement of metabolic rate in *Drosophila* is sex. There is considerable sexual dimorphism in *Drosophila*, with females being as much as 40-50% heavier than males (Piper et al., 2014). Yet, the majority of studies have tended to ignore sex in their experimental groups or, alternatively, have used either all females or all males in their metabolic measurements. These approaches either obscure the effects of social interactions when multiple flies are assessed, or eliminate them when single flies are subject to experimentation.

Given the key importance of *Drosophila* as an animal model, and the prominence of metabolic rate measurements in current studies, there is a compelling need to understand biotic sources of variation in measurements of metabolic rate. Consequently, we have measured oxygen consumption in *D. melanogaster* independently in males and females, and as a function of density (number of flies per respirometer chamber). Specifically, we hypothesized that oxygen consumption in *D. melanogaster* would be influenced by both social interactions and by sex. We additionally measured P_crit_, the partial pressure of oxygen at which oxygen consumption begins to decline, as this variable is an indicator of hypoxic tolerance.

## RESULTS

### Body mass

**Fig. 1.**
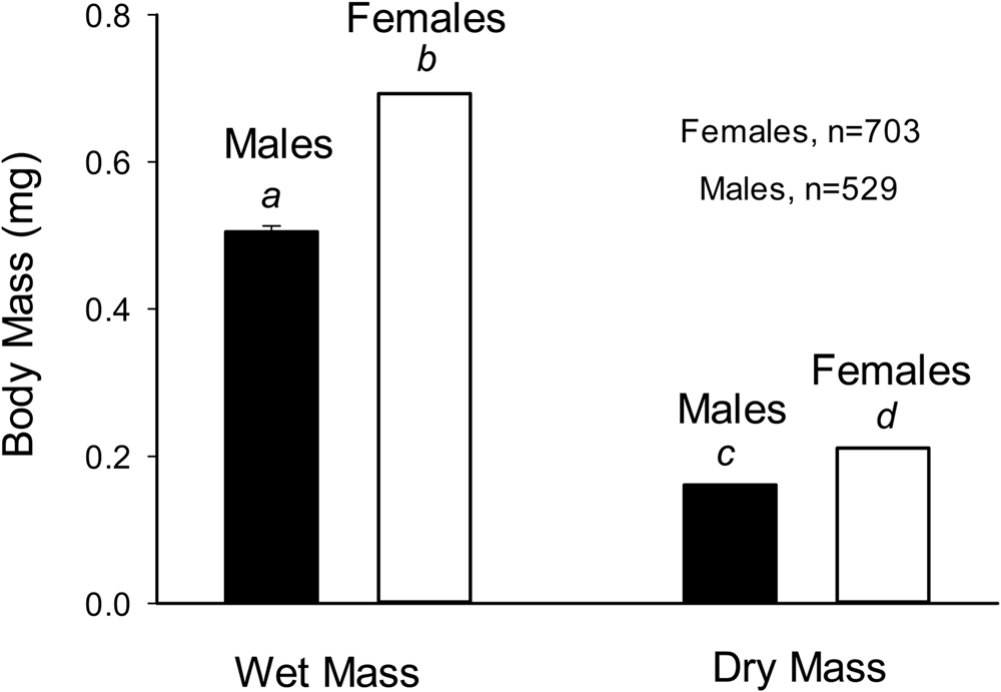
**Wet and dry body mass in female and male *D. melanogaster.*** Mean±s.e. are plotted, but standard errors are too small to be visible (see text). *N*=703 for females, 529 for males. Different lower case italic letters indicate significance differences between groups at the *P*<0.01 level (*t*-test).

Wet body mass was significantly higher (*P*<0.02) in adult female flies (0.693±0.050 mg) compared with adult male flies (0.505±0.047 mg) (Fig. 1). Similarly, dry body mass was significantly higher (*P*<0.009) in adult female flies (0.211±0.012 mg) compared with adult male flies (0.161±0.008 mg).

### Mass-specific routine oxygen consumption

**Fig. 2.**
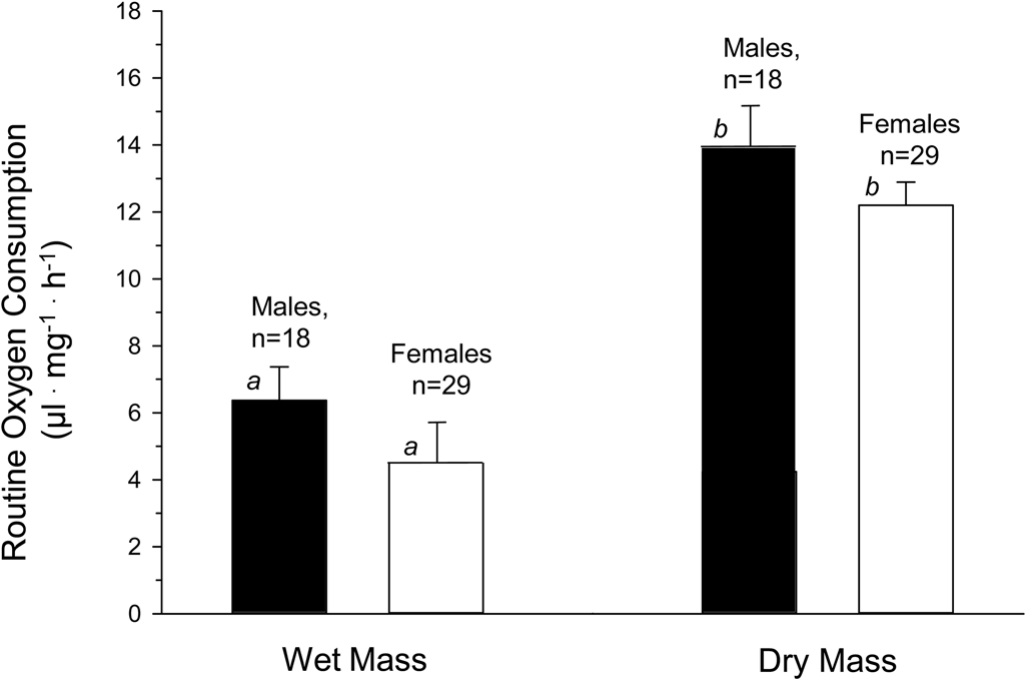
**Wet and dry mass-specific routine oxygen consumption (*V̇O*_2_) in male and female *D. melanogaster*.** Mean values±s.e. are presented. Also indicated are *n* values, where each value contributing to *n* is a separate trial comprising multiple flies in each respirometer. The average number of flies in the respirometer (density) for *n* runs, was 18±3 for males and 29±3 for females. Different lower case italic letters indicate significance differences between groups at the *P*<0.01 level (*t*-test).

Mass-specific *V̇O*_2_, based on either wet or dry mass, are shown for male and female adult *D. melanogaster* in Fig. 2. *V̇O*_2_ expressed for wet mass in males and females was ∼6.0 and 4.5 µl O_2_ mg^−1^ h^−1^, respectively. There was no significant difference (*P*>0.05) between *V̇O*_2_ in males and females, based on either wet or dry mass, when pooling all respirometry data irrespective of density of flies in the respirometer.

**Fig. 3.**
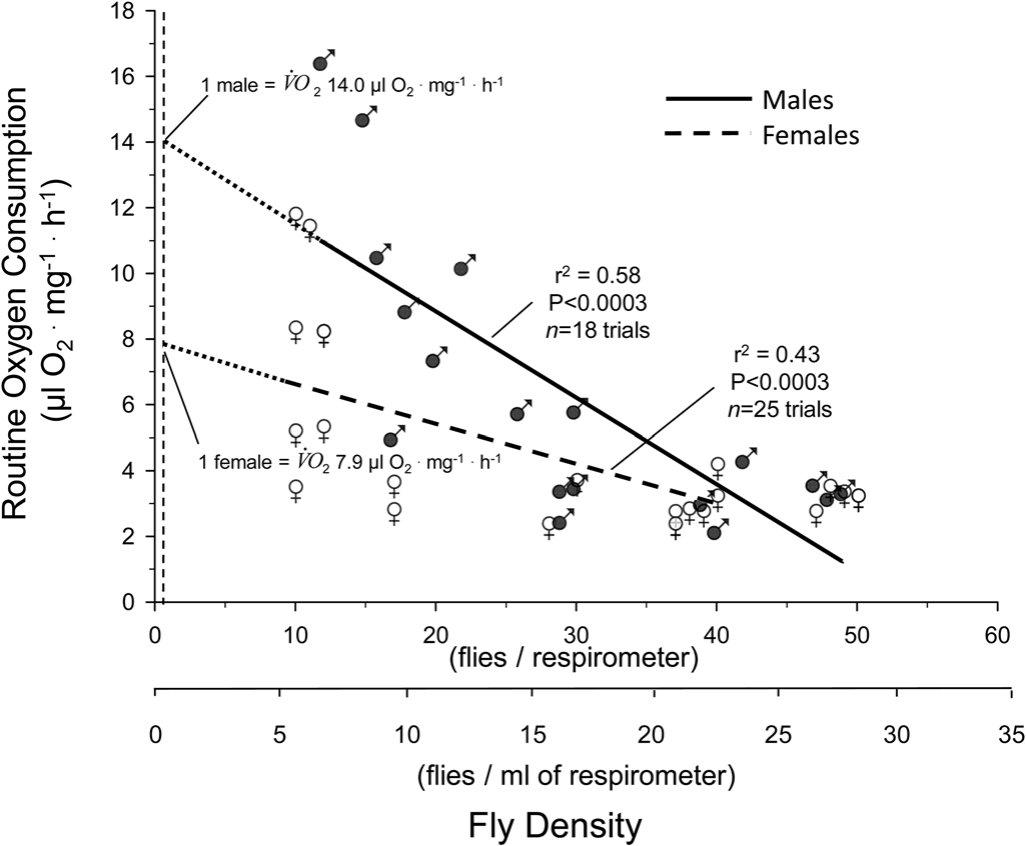
**Mass-specific routine *V̇O*_2_ in normoxia as a function of density in male and female *D. melanogaster*.** Density is shown on both a per respirometer and per ml of respirometer basis. Separate linear regressions for male (*n*=18) and female (*n*=25) flies are provided, along with the correlation coefficient and *P* value for each. Dashed lines represent the extrapolation of each relationship back to the value for a single fly. The slope of the two lines was significantly different (*P*<0.02, least squares method).

This general approach of pooling all data obfuscates sex differences that emerge when also controlling for fly density. Fig. 3 shows the relationship between mass-specific *V̇O*_2_ as a function of fly density within the respirometer. Both male and female flies exhibited greatly decreased *V̇O*_2_ as fly density increased. *V̇O*_2_ decreased to just 15% and 40% in males and females, respectively, as fly density increased from 10 to 50 flies·respirometer chamber^−1^. Assuming a linear relationship, extrapolation of the relationship between density and *V̇O*_2_ back to density of one fly per respirometer indicated a profound sex-based difference in *V̇O*_2__,_ with a hypothetical single male having a *V̇O*_2_ of ∼14 µl O_2_ mg^−1^ h^−1^ but only ∼8 µl O_2_ mg^−1^ h^−1^ in a single female. The sex-dependent difference in the *V̇O*_2_ relationship is underscored by the fact that the regressions relating *V̇O*_2_ to density in males and females had significantly different slopes (−0.27 males, −0.12 females; *P*=0.017) and intercepts (13.87 males, 7.84 females; *P*=0.001). Importantly, these decreases in metabolic rate are not a function of decreased PO_2_ within the respirometers related to crowding – all data reported in Fig. 3 were recorded in a ‘normoxic’ PO_2_ above 16 kPa.

Noteworthy is that extrapolation of these *V̇O*_2_ values back to the origin to predict values for single flies is based on a highly significant (*P*<0.003) linear regression. Use of a quadratic equation to describe the existing data produces a similarly high level of significance (*P*<0.001), but does not render realistic values when extrapolated back beyond the existing data towards the origin for determination of values for individual flies.

### Critical partial pressure (P_Crit_)

**Fig. 4.**
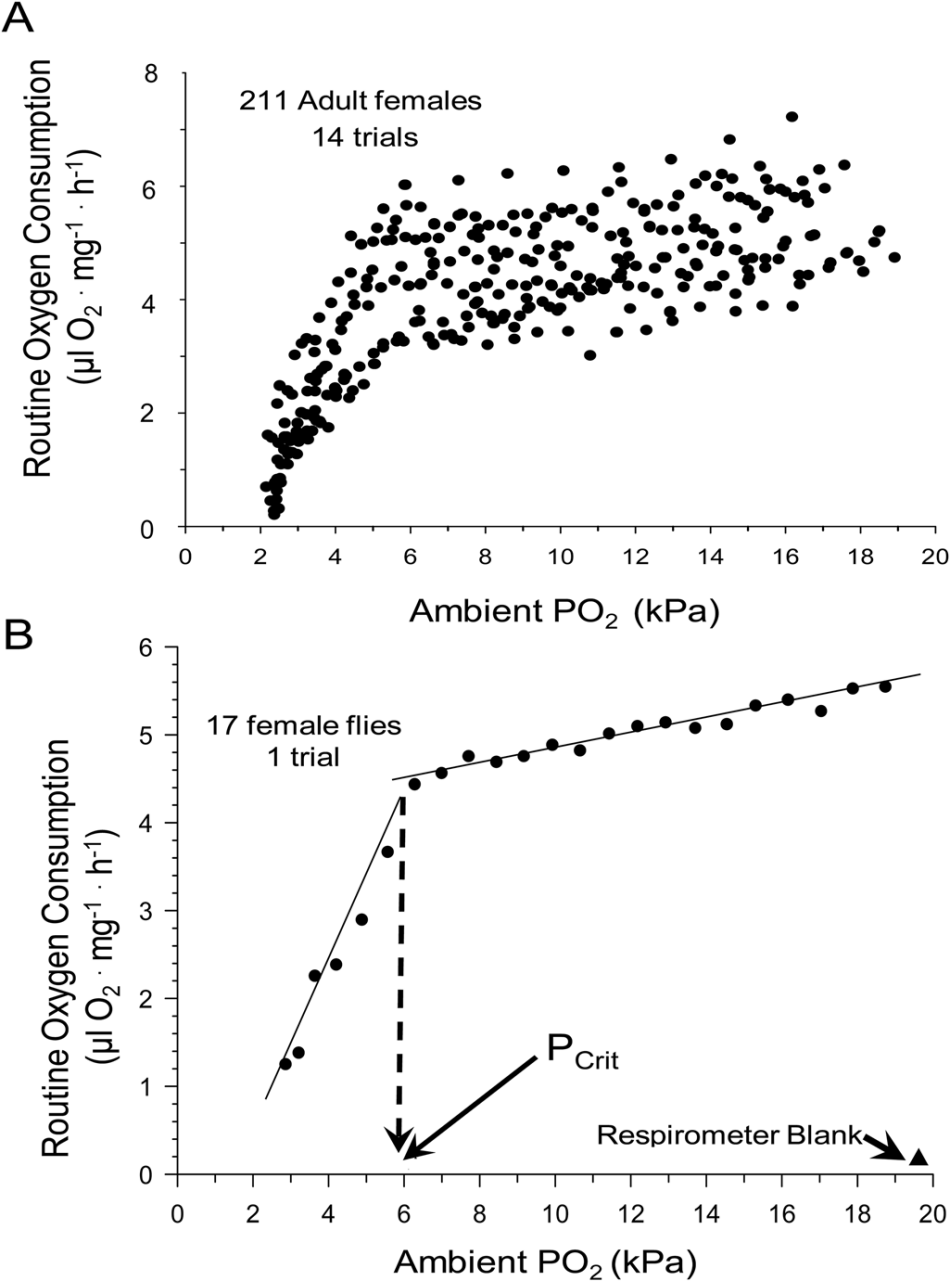
**P_Crit_ determination in adult *D. melanogaster.*** (A) A scatter plot of 11 P_Crit_ trials (211 females in total). (B) Representative routine *V̇O*_2_ data derived from a single experimental trial of a respirometer containing 17 female flies. Also shown are the values for a respirometer blank. The intersection of the two linear regressions indicates the P_Crit_ value, identified on the X-axis by the vertical dashed arrow (see text for details of P_Crit_ calculation and statistics).

Critical partial pressure (P_Crit_) was determined in 14 separate trials involving 211 flies of both sexes. Fig. 4A shows a scatterplot of the acquired data, indicating that most flies ceased to consume oxygen at around 2 kPa. Fig. 4B shows a representative trial of 17 female flies, yielding a P_Crit_ of 5.9 kPa for this respirometry trial.

**Fig. 5.**
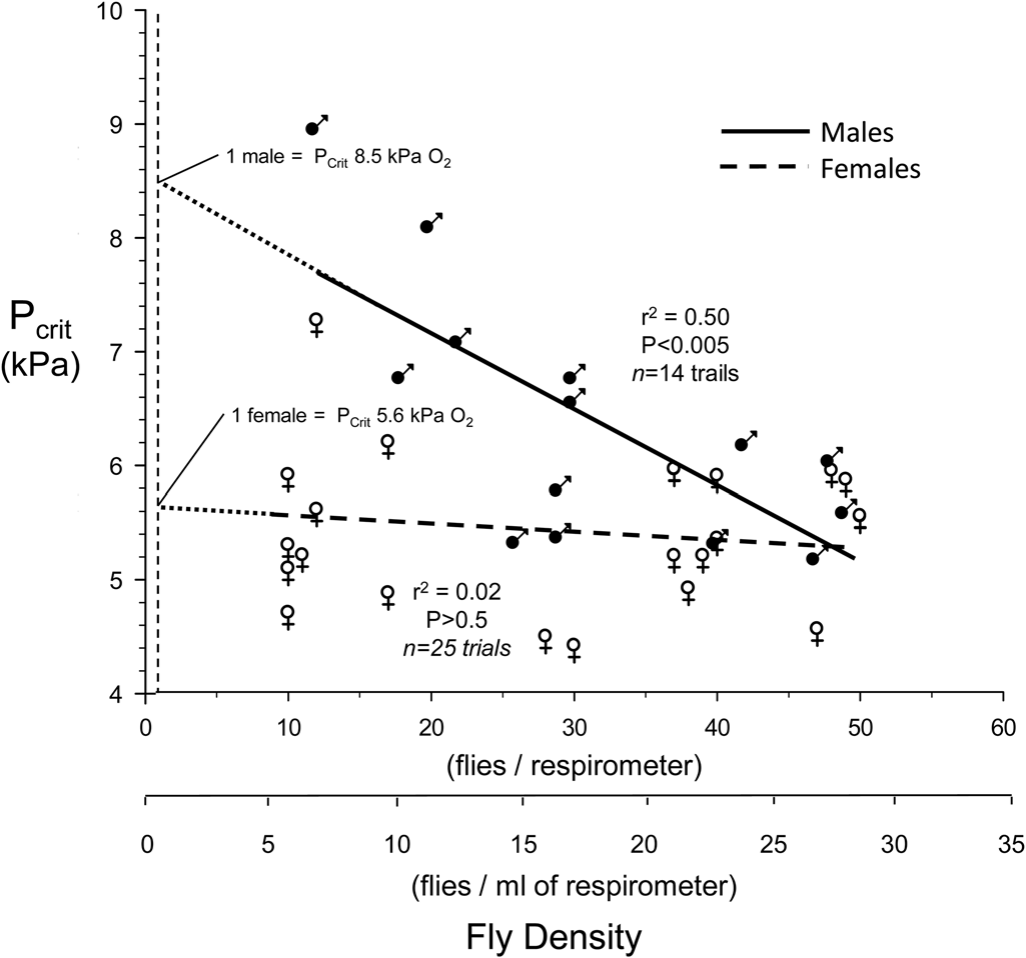
**P_Crit_ as a function of respirometer fly density in male and female *D. melanogaster.*** Density is shown on both a per respirometer and per ml of respirometer basis. Separate linear regressions for male (*n*=14) and female (*n*=25) flies are provided, along with the correlation coefficient and *P* value for each (least squares method). Dashed lines represent the extrapolation of each relationship back to the value for a single fly. The slope of the line for female flies was not significant (*P*>0.05), indicating that P_Crit_ in females as lines was unaffected by fly density.

P_Crit_ as a function of fly density in the respirometry chambers is shown in Fig. 5. P_Crit_ in female flies was ∼5-6 kPa, with the extrapolated value for a single fly being 5.6 kPa. For females, density had no significant effect on P_Crit_ (*P*>0.05); however, P_Crit_ was highly dependent upon fly density for males (*P*<0.005), with values ranging from ∼5.5 kPa at 50 flies chamber^−1^ up to an extrapolated value of 8.5 kPa for a single fly per chamber.

**Fig. 6.**
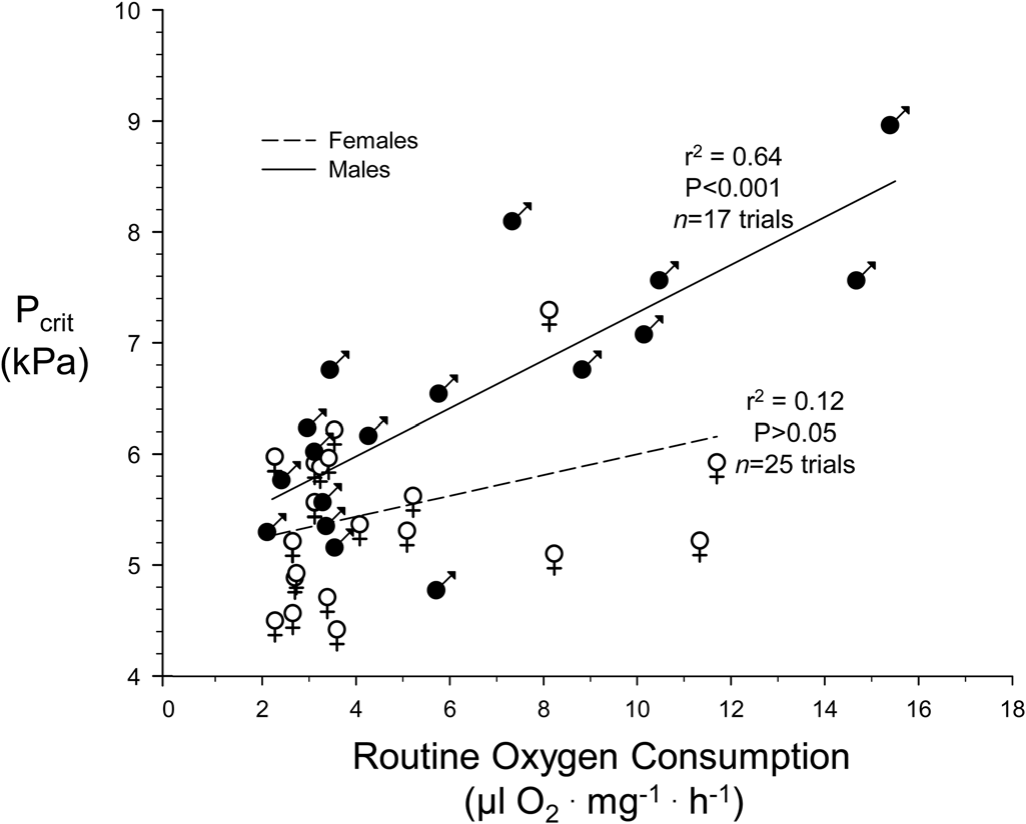
**Relationship between P_Crit_ and routine *V̇O*_2_ in male and female *D. melanogaster*.** *N*=17 trials for males and 25 trials for females (least squares method). Average fly density of all trials was 29±2 flies respirometer^−1^.

Reflecting the same sex-related patterns for P_Crit_ and fly density, the relationship between P_Crit_ and *V̇O*_2_ was not significant in females (*P*>0.05) but highly significant in males (*P*<0.001). Thus, in males with a higher routine *V̇O*_2_, P_Crit_ was correspondingly higher (Fig. 6).

### Prandial effects on routine *V̇O*_2_ and P_Crit_

The effects of 24 h of fasting on *V̇O*_2_ and P_Crit_ values in *D. melanogaster* fed *ad libitum* and fasted for 24 h are provided in Table 1. A *t*-test indicated that fasting to this extent had no significant effect on either variable.

**Table 1.**
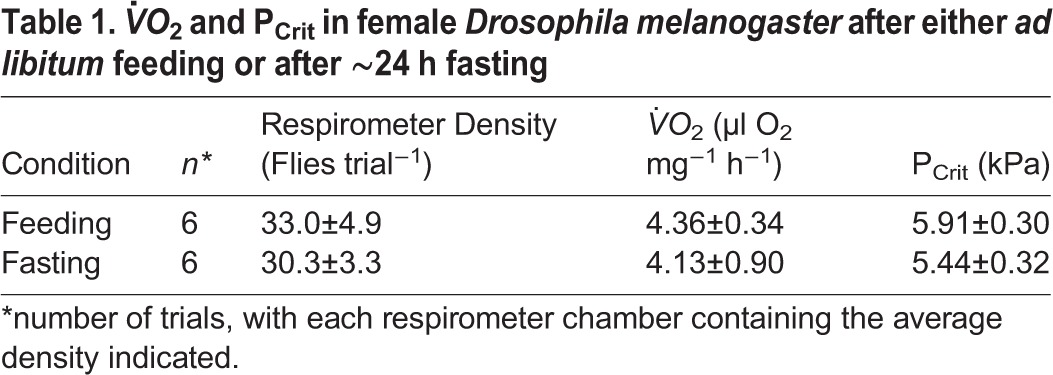
***V̇O*_2_ and P_Crit_ in female *Drosophila melanogaster* after either *ad libitum* feeding or after ∼24 h fasting**

## DISCUSSION

Interpretation of the experimental data from the present study is best done against the backdrop of the extensive, yet highly variable and contradictory, literature on the metabolic rate of *Drosophila* spp*.* Consequently, we have assembled published data for this species (Table 2). Noteworthy is that not all listed studies reported temperature, gender or sufficient data to calculate fly density in the respirometers during oxygen consumption measurement, preventing a robust meta-analysis.

**Table 2.**
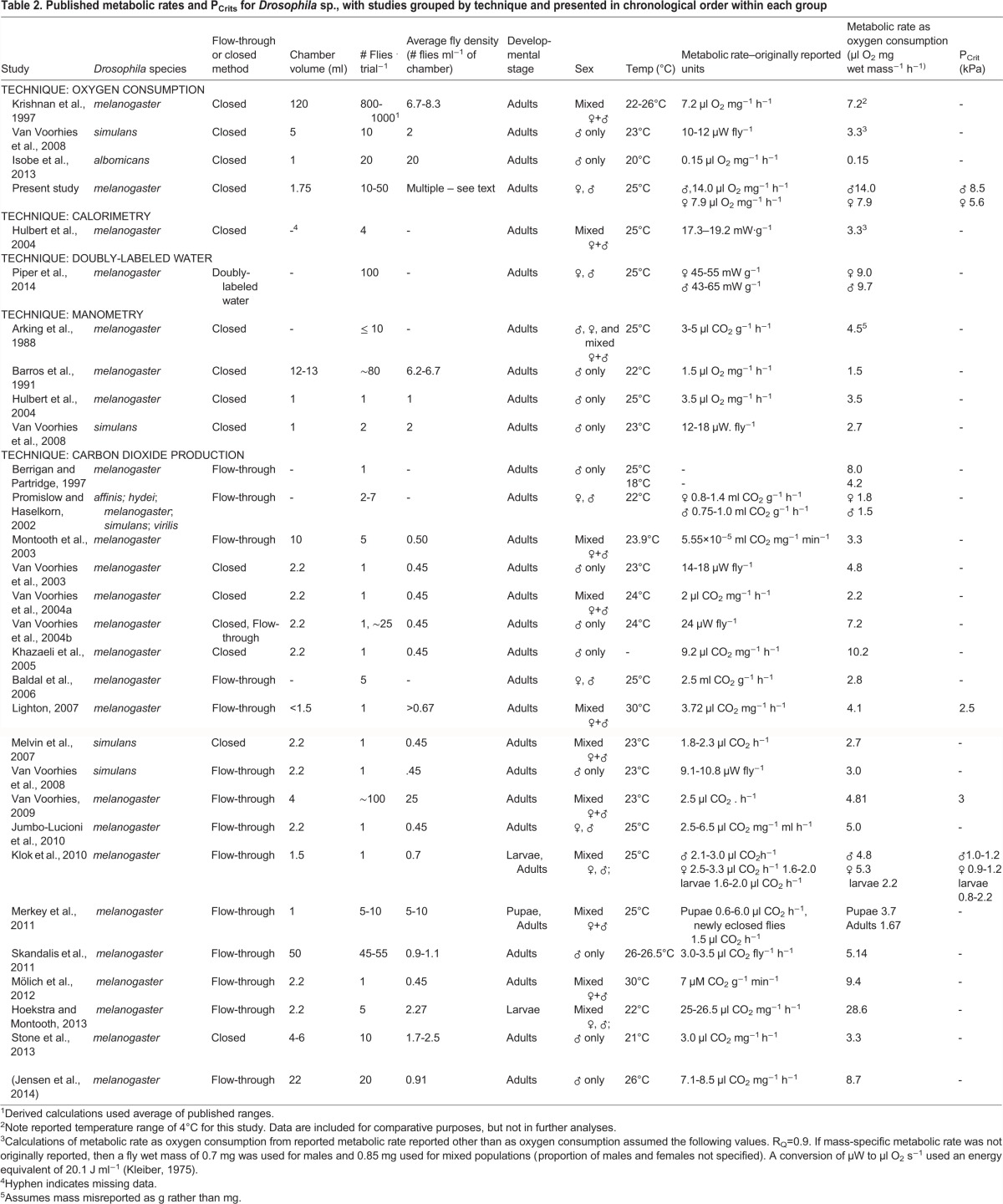
**Published metabolic rates and P_Crits_ for *Drosophila* sp., with studies grouped by technique and presented in chronological order within each group**

### Sex and oxygen consumption

The specific influence of sex on metabolic rate is not clear from our survey of the literature (Table 2). A relatively early study of the responses of *D. melanogaster* to anoxia concluded that there were no physiological differences between sexes (Krishnan et al., 1997). In contrast, while statistics were not explicitly given, the metabolic rate (measured as CO_2_ production) of isolated individual female flies appeared to be anywhere from 30-40% higher (Van Voorhies et al., 2004b) to 200-300% higher (Van Voorhies et al., 2004a) than males under the same conditions. Another study indicates that there is no significance difference in *V̇O*_2_ between male and female flies measured at a density of 1 fly ml^−1^ of respirometer (Klok et al., 2010). The present study suggests that at higher respirometer densities there is little or no difference between oxygen consumption in males and females, but there is a significant effect of density such that as fly density declines, males begin to exhibit higher metabolic rates than females (Fig. 3). The discrepancy among studies may be due to the interaction of sex and factors such as rearing condition (mixed sex, density, food source), fly age, or female condition (virgin versus non-virgin). In aggregate, only about one half of the studies identified in our survey (Table 2) actually control for sex, with many studies using unsorted mixtures of male and female flies. Clearly, future studies that control for and investigate sex of the flies are called for.

### Critical PO_2_ for oxygen consumption

The critical PO_2_ for oxygen consumption, P_Crit_, provides an indication of the ability of the animal to maintain its *V̇O*_2_ as ambient PO_2_ declines, and is often viewed as an indicator of tolerance to acute hypoxia. P_Crit_ is much less frequently measured than metabolic rate itself because of the necessity of a more involved experimental protocol. Indeed, our survey revealed that P_Crit_ values were measured in only four studies of *Drosophila* (including the present study), averaging 3.4±1.3 kPa. P_Crit_ values for insects vary widely, but generally fall in the range of 2-8 kPa (Chown and Nicolson, 2004; Greenlee and Harrison, 2004; Harrison et al., 2006; Harrison and Roberts, 2000; Hoback and Stanley, 2001; Lease et al., 2012; Owings et al., 2014). In the current study, P_Crit_ for single flies, determined from extrapolation, was ∼5.6 for females and 8.5 for males. These values, while in line with published values for other insects, are higher than mean value of ∼3.4 kPa derived from the few values revealed in our survey and much higher than the very low estimates of P_Crit_ of less than 1 for adult *D. melanogaster* measured by Klok and his collaborators (Klok et al., 2010) (Table 2).

One critique of the current methodology of the current for measuring P_crit_ was that it required several hours in a closed respirometer for P_Crit_ determination. It is possible that during that measurement period the beginning of desiccation and/or starvation could have begun influencing oxygen consumption or other physiological variables, since these are potentially profound stressors in *Drosophila* (Gibbs, 2002; Hardy et al., 2015; Masek et al., 2014; Rajpurohit et al., 2016). Consequently, we wish to emphasize not the overall P_Crit_ values for adult *D. melanogaster* determined in our study, but rather the fact that the values in the present study were dependent on fly density, which is a novel observation. Additionally, the current study is among the first on *Drosophila* to examine sex-dependence of P_Crit_ in insects. Here we report a large difference in P_Crit_ estimated for individual flies in males (8.5 kPa) compared to females (5.6 kPa). Males not only had a higher P_Crit_ but also showed a sharply decreasing P_Crit_ as fly density in the respirometer increased (Fig. 5). The mechanism underlying these pronounced sex-based differences is not clear. The females of *Drosophila* are generally larger than males (Fig. 1), which might be thought to lead to slighter larger diffusion distances for O_2_ within the tracheal system; however, this would lead to higher, rather than lower, P_Crit_ in females. In any event, a systematic examination of the literature has indicated that P_Crit_ in insects is independent of body mass (Harrison et al., 2014; Lease et al., 2012). Another possibility would arise if males have intrinsically higher levels of *V̇O*_2_ which are correlated with a higher P_Crit_ (Fig. 6). Yet, in aggregate, there is no significant difference in *V̇O*_2_ between males and females (Fig. 2). Clearly, further experimentation is required to verify these findings and determine the basis for sex-based differences in P_Crit_ in *Drosophila*.

### Density-dependence of oxygen consumption *V̇O*_2_ and P_Crit_

Density of flies in respirometers, like sex of the flies examined, varies widely in the literature, with densities ranging from 1-100 flies per respirometer (Table 2). New methodologies with greater sensitivities for measuring metabolic rate, especially using CO_2_ production, are now enabling metabolic rate measurements in individual flies, which has become the new norm in the last decade (Table 2). This protocol very closely mirrors that used for decades (truly, for centuries) for larger animals, which are typically measured individually, in part because this protocol streamlines the calculations of mass-specific *V̇O*_2_. Indeed, the ability to measure metabolic rate in individual flies has been promoted as an advancement “which greatly increases the sample size and statistical power of experimental studies and allows the effects of individual differences in body size to be taken into account” (Van Voorhies et al., 2004b). Indeed, most studies are now done on individual flies (Table 2).

What may be the consequences of social isolation to the measurement of metabolic rate in *Drosophila*? Clearly, measuring metabolic rate in an individual fruit fly (or ant or other small social insect) is a technical *tour de force.* Yet at least in the present study a protocol that varies fly density in the respirometer profoundly affects both metabolic rate and P_Crit_. Although females are less sensitive to the effects of fly density than males, in both sexes the estimated routine mass-specific *V̇O*_2_ of an individual, as estimated from extrapolation from multiple fly groups, is much higher than groups of flies. A single previous study that we could identify has investigated the ‘group effect’, investigating the difference between metabolic rate measured in individual flies and in groups of 20 individuals, and reported no difference as a function of density (Van Voorhies et al., 2004b). That study also reported, perhaps not surprisingly, that actual physical confinement (entrapment with cotton balls) elevated metabolic rate by as much as 50%. However, even at the highest densities of ∼50 flies per respirometer in the current study, there was no forced physical contact between flies, nor any physical confinement within the respirometer itself. Importantly, the densities used in the present experiments designed to measure routine oxygen consumption are unlikely to have resulted in any depression of metabolism from the development of hypoxia, because all respirometer runs for routine oxygen consumption were completed after a decline in PO_2_ of just a few kPa and well before PO_2_ levels fell below P_Crit_. Another factor to be considered in contemplating density effects on routine oxygen consumption involves a possible effect of enhanced male aggression, which might lead to elevated rates of oxygen consumption. Yet, one could reasonably expect such aggression to increase as fly density in the respirometers increased, but our findings show quite the opposite effect where increased density leads to reduced routine oxygen consumption.

What, then, could be the mechanism for these high metabolic rates in individual flies or flies in smaller groups from the present study? One explanation is that the high metabolic rates we extrapolate for individual flies represent flies experiencing stress as a result of their social isolation. This begs the question of what is a ‘routine’ metabolic rate for a social insect, that of an individual out of its social context, or that while interacting normally with other individuals? Adult fruit flies are involved in a multitude of social behaviors that often surround courtship and aggression (Eban-Rothschild and Bloch, 2012; Herrero, 2012; Kravitz and Fernandez Mde, 2015; Pavlou and Goodwin, 2013; Villella and Hall, 2008; Yamamoto et al., 2014). While we may think of courtship and aggression as social behaviors that elevate metabolic rate, social isolation of individuals is not normal for fruit flies and may lead to stress (and thus an elevated metabolic rate) equal to or greater than that resulting from normal social behaviors between flies. Indeed, there is a long-established literature, including that oriented towards ‘animal well-being’, e.g. Hirata et al. (2016) and Lyons et al. (1993), that suggests that social isolation can directly elevates physiological rates. This is a broadly based finding for schooling fishes (Nadler et al., 2016; Parker, 1973; Schleuter et al., 2007), birds (Khan et al., 2015; Soleimani et al., 2012) and mammals (Krause and Ruxton, 2002; Martin et al., 1980; Rushen et al., 1999). Generally, the hypothalamic-pituitary-adrenocortical (HPA) system is implicated in these responses (Hennessy et al., 2009; Hernandez et al., 2010; Herskin et al., 2007). Stress-related phenomena associated with social isolation are also being explored in insects, including *Drosophila* (Kohlmeier et al., 2016; McNeil et al., 2015; Soleimani et al., 2012; Ueda and Wu, 2009), where both short- and long-term influences of isolation on aggressive behavior and basic physiological processes (e.g. neuromuscular excitability, altered cellular ROS regulation) are being revealed. Flies are presumably carrying out interactive behaviors in any study with multiple individuals per respirometer. Olfactory cues are important to the behavior of *Drosophila* (Eban-Rothschild and Bloch, 2012), with flies likely responding to pheromones or other environmental cues in a respirometer. It is unclear whether this ‘pheromone hypothesis’ would be affected by measurements that use either closed or flow-through (‘open’) respirometry. Given the potency of insect pheromones, including those of *Drosophila* (Kohl et al., 2015), and the very low flow rates of most flow-through respirometry, it is likely that multiple flies in a respirometer are exposed to pheromones with either technique. Ironically then, the increasing trend to measure metabolic rate in individual fruit flies, in part because we now can, has artificially removed these animals from their normal social context involving a multitude of behavioral interactions largely driven by the release, reception and perception of pheromones and visual and tactile clues (Dweck et al., 2015; Piper et al., 2014; Schneider et al., 2016; Vijayan et al., 2014).

Although beyond the scope of the present experiments, worth mentioning is that consideration of fly density is typically in the context of the individual. Yet, there may be implications of density variation and metabolic rate at the level of the colony in social insects. For example, behavioral organization in colonies of the seed-harvester *Pogonomyrmex californicus* affects metabolic rate and alterations in growth patterns at the colony-level (Waters et al., 2010). How density within the colony plays into the allometric scaling, activity and growth of whole colonies of social insects will be a highly interesting focus of future studies.

Finally, fly density goes beyond effects on metabolism. Experiments raising larval *Drosophila* under various group densities discovered an inverse exponential relationship between group density and nervous system morphology, including synaptic bouton numbers, as well as the number and length of axonal branches (Stewart and McLean, 2004). Additionally, there was a direct density-dependence of concentration of Fasciclin-II, a cell membrane glycoprotein important in the process of axonal fasciculation.

Further experimentation is warranted to determine the full extent of group density on the biology of *Drosophila*, and especially to determine metabolic rate in the presence and absence of cues associated with social behaviors. Additional future experiments on density effects should also establish whether the relationship between respirometer fly density and *V̇O*_2_ is in fact a U- or V-shaped curve, with higher densities than used in the current study leading to stress-related increases in *V̇O*_2_.

### Oxygen consumption and prandial state

The present study discovered no significant difference between adult *Drosophila* feeding *ad libitum* and flies starved for 24 h. Similarly, a fasting period of 4 h had no effect on metabolic rate, although the R_Q_ value was decreased by fasting (Van Voorhies et al., 2004b). Collectively, these data suggest that there is little or no specific dynamic action (SDA) in either the short- or long-term. In contrast to these findings, Baldal et al. reported lowered metabolic rate as a result of starvation but, in the long-term across generations, elevated metabolic rates associated with starvation resistance (Baldal et al., 2006). Again, variation exists in the literature, with another long-term study reporting no change in metabolic rate produced by chronic diet restriction (Hulbert et al., 2004). Interestingly, starvation has no effect on the respiration of mitochondria isolated from fasting adults (Partridge et al., 2005). Clearly, more experiments are required to understand the full interactions between metabolic rate and prandial state in *Drosophila*.

A complex set of interactions occurs between various activities, desiccation, starvation/feeding and numerous physiological processes (Hardy et al., 2015; Masek et al., 2014; Rajpurohit et al., 2016; Slocumb et al., 2015). How fly density may interact with these variables is currently not well understood. Future studies to both tease apart these factors as well as explore synergies are highly warranted.

### Conclusions

This study is the first to systematically report on the effect of density (and thus social interaction) on the metabolic rate and critical O_2_ partial pressure of *Drosophila.* Highly significant effects of respirometer fly density are evident for both physiological parameters. Values of *V̇O*_2_ determined in the present study are within the range of those previously published for *Drosophila*. Our findings, plus examination of previously published data (Table 2), suggests that greater attention be paid to controlling for (and reporting) not only respirometer density, but also sex, temperature and genetic strain.

## MATERIALS AND METHODS

### Maintenance and identification

Fruit flies *D. melanogaster* (Meigen, 1830) (wild-type, Oregon R strain, Carolina Biological Supply Company, Burlington, NC, USA) were grown and maintained at 25°C in a 14 h light:10 h dark photoperiod. Flies were kept in standard densities of ∼150 flies per 250 ml volume plastic vial, with ∼30 ml of standard *Drosophila* medium plus Baker's yeast (Formula 4-24 Instant Drosophila Medium, Carolina Biological Supply Company) in the bottom of the rearing bottle. *Drosophila* stock was maintained by transferring flies to new culture vials every 10-14 days. For experiments, ∼30 mixed-sex adult flies from stock culture were placed into a fresh vial on day 0. On day 2, the adult flies were removed from the vials, and eggs laid in the vials were allowed to develop into adult flies. On day 12, adult flies were transferred without anesthesia to fresh vials. Thereafter, adult flies were transferred every sixth day to fresh vials until assayed.

Metabolic rate of 5-day-old adult flies is significantly higher than that at 16, 29 or 47 days of age, with these three older stages not being significantly different from each other (Van Voorhies et al., 2003). Consequently, we restricted our measurements to adult flies 10-20 days old. Flies were anesthetized with 2-3 min of exposure to FlyNap^®^ (Carolina Biological Supply Company) prior to sorting for sex determination. Males were identified based on the presence of sex combs. Males and females were kept separated and allowed to recover from anesthesia with free access to food for at least one day prior to oxygen consumption measurement. However, one group was fasted for 24 h prior to metabolic measurements (see below).

### Body mass

Immediately following measurement of oxygen consumption (see below), flies were anesthetized with FlyNap^®^ and wet mass (mg) determined to the nearest 0.01 mg. Flies were then placed in a 60°C oven to dry for 24 h prior to measuring dry mass (mg).

### Measurement of routine oxygen consumption

Routine oxygen consumption (*V̇O*_2_) was measured at 25°C on individual groups comprising a known number of flies, ranging from 10 to 50 individuals. Each group of flies was placed in a polypropylene centrifuge tube with the following specifications: (8.2 mm diameter, 35 mm length, 0.9 mm wall thickness) sealed with a polypropylene cap of identical thickness. Net respirometer chamber gas volume was 1.751±0.002 ml (*n*=15), as measured by weighing the water-filled respirometer at 25°C and using water density to determine volume. Given the very small variation in respirometer gas volume from assembly to assembly, a respirometer gas volume of 1.751 ml was used in all *V̇O*_2_ calculations. Each chamber contained 50 mg of soda lime pellets for CO_2_ absorption, which were kept separate from the flies by a small wad of cotton batting (soda lime also absorbs water vapor, so flies were likely subjected to some small degree of desiccation especially during the longer *V̇O*_2_ trials, which was not controlled for). The volume of the cotton and soda lime was determined for each run, and subtracted from the respirometer volume for each measurement. All respirometers were submerged in a water bath maintained at 25°C±0.2°C during the *V̇O*_2_ measurements.

Flies were allowed a 30 min acclimation period in the chamber, which was gently flushed with fresh air during this period. The respirometer chamber was then sealed, and a fiberoptic O_2_ detection probe (see below) was gently inserted through a 1 mm gas-tight orifice in the center of the respirometer cap, and advanced to the center of the respirometer. A blank was created by an identically treated respirometer chamber without flies. Respirometer chambers were then gently placed into a 25°C covered water bath, where they remained for the duration of the *V̇O*_2_ measurement period. Experiments were carried out in the darkened respirometers.

PO_2_ in the respirometer chamber was recorded in real time using a FOXY 40 Hz O_2_ probe attached to a MultiFrequency Phase Fluorometer (MFPF-100) system made by OceanOptics, Inc. (Dunedin, FL, USA). The output of the system was attached to a computer running Tau Theta software (OceanOptics Inc.). Response time of the probe was <1 s. The probe was subjected to a three point calibration with dried gases at a PO_2_ of 1, 10 and 20 kPa. Preliminary experiments revealed that the movements of the flies themselves (body movements, wing movements) created sufficient gas convection currents within the respirometer to keep the gas within sufficiently mixed, so no potentially disturbing additional gas mixing within the chambers was provided by the researchers.

Given the small oxygen consumptions of the flies, any significant inward diffusion of oxygen across the respirometer walls, or leakage of water through the respirometer lid, could affect the accuracy of the measurements. Thus, to verify the suitability of the respirometers for these experiments, permeability measurements were made on six empty respirometers to determine the rate of inward O_2_ diffusion from the surrounding water. The respirometers were filled with 100% N_2_ gas and then submerged in the water bath. The subsequent increase in PO_2_ within each respirometer was then measured at the end of 2.5 h of submergence. No water was detected leaking into any of the respirometers. The rate of PO_2_ increase in the submerged respirometers over the submersion period was only 0.0021±0.007 kPa h^−1^ kPa^−1^ of inward PO_2_ pressure gradient driving diffusion (mean±standard error, *n*=6), even with an inward partial pressure gradient across the wall of ∼20 kPa. As described below, 5 h was the longest time period that any flies spent in the respirometers and that the lowest PO_2_s at the end of that 5 h period were ∼2 kPa. Thus, the respirometer testing was under conditions as lengthy and with an inward diffusion pressure gradient larger than would exist under even the most extreme O_2_ depletion occurring during actual respirometer runs. Consequently, we concluded that the respirometers were functionally both O_2_ impermeable and leak-proof, and no corrections were necessary for the subsequent *V̇O*_2_ calculations.

### Experimental protocols for routine *V̇O*_2_ and critical partial pressure

The first series of respirometry runs (trials) were designed to measure routine *V̇O*_2_. PO_2_ was allowed to fall from atmospheric (∼20 kPa) to no lower than ∼16 kPa during these runs, generally requiring 1-2 h, depending on fly density in the respirometers (greater density required less time for O_2_ depletion). This level of O_2_ was well above the PO_2_ at which oxygen consumption began to be affected by ambient PO_2_.

The second series of runs determined critical oxygen partial pressure, P_Crit_ (expressed in kPa), essentially the ambient PO_2_ below which an animal's *V̇O*_2_ begins to decline, that is, below which *V̇O*_2_ cannot be maintained. For these experiments, flies were kept in the respirometer until PO_2_ had decreased to severely hypoxic levels, typically to a point at which no further changes in PO_2_ were detected, a process typically taking 3-5 h in total (the 1-2 h to reach the P_Crit_ plus the additional 2-4 h to move to a PO_2_ well below P_crit_). Again, the time for depletion varied greatly, as P_Crit_ was determined over a fly density range of 10 to 50 flies per respirometers.

### Prandial state and routine oxygen consumption

In an experiment designed to consider the effect of prandial state on *V̇O*_2_ and hypoxia tolerance, female flies were separated into two groups, one with *ad libitum* access to food prior to placement in the respirometer, while the others were starved, with free access to water via moistened filter paper, for 18 h prior to *V̇O*_2_ measurement. Changes in lipid and other metabolic pathways occur in as little as 4-6 h of fasting in *Drosophila* (Chatterjee et al., 2014; Choi et al., 2015), with 24 h of fasting being sufficient to cause major biochemical disturbances (Menger et al., 2015; Park et al., 2014). Thus, 24 h of fasting was viewed as a strong stressor without creating a morbid metabolic physiology that accompanies >30-40 h starvation at 25°C. Flies from feeding and fasting groups were then placed in the plastic respirometers and their *V̇O*_2_ and P_Crit_ determined as indicated above.

### Routine oxygen consumption calculation

Mass-specific oxygen routine consumption, *V̇O*_2_, was calculated from the chamber volume (minus the volume of the soda lime pellets, cotton batting and estimated volume of the flies), the rate of decline of PO_2_ in the respirometer chamber, and the total mass of the flies in the respirometer, and was expressed as µl O_2_ mg^−1^ h^−1^. All *V̇O*_2_ values are expressed on a wet mass basis unless indicated otherwise.

### Statistics

Differences in *V̇O*_2_ between males and females (independent of fly density) were tested with separate *t*-tests for wet and dry body mass calculations. Linear regressions were generated and tested for significance of routine oxygen consumption as a function of fly density and sex. P_Crit_ was then determined for each respirometry run using a MATLAB program designed to analyze critical inflection points in the relationship between ambient PO_2_ and *V̇O*_2_ (Yeager and Ultsch, 1989). The slopes and intercepts of regression lines were compared for significant difference using Student's *t*-test. A significance level of 0.05 was adopted for all statistical tests, which were performed using SigmaPlot (San Jose, CA, USA) and Statistica (Dell Statistica, Tulsa, OK, USA) statistical software.
